# Prolonged Corrosion Stability of a Microchip Sensor Implant during *In Vivo* Exposure

**DOI:** 10.3390/bios8010013

**Published:** 2018-02-01

**Authors:** Paul Glogener, Michael Krause, Jens Katzer, Markus A. Schubert, Mario Birkholz, Olaf Bellmann, Claudia Kröger-Koch, Harald M. Hammon, Cornelia C. Metges, Christine Welsch, Roman Ruff, Klaus P. Hoffmann

**Affiliations:** 1 IHP, Im Technologiepark 25, 15236 Frankfurt (Oder), Germany; 2 Leibniz-Institut für Nutztierbiologie (FBN), W. Stahl Allee 2, 18196 Dummerstorf, Germany; 3 Fraunhofer Institut für Biomedizinische Technik, Joseph-von-Fraunhofer-Weg 1, 66280 Sulzbach, Germany

**Keywords:** implant, biostability, semipermeable membrane, MEMS, CMOS

## Abstract

A microelectronic biosensor was subjected to *in vivo* exposure by implanting it in the vicinity of *m. trapezii* (Trapezius muscle) from cattle. The implant is intended for the continuous monitoring of glucose levels, and the study aimed at evaluating the biostability of exposed semiconductor surfaces. The sensor chip was a microelectromechanical system (MEMS) prepared using 0.25 µm complementary metal–oxide–semiconductor CMOS/BiCMOS technology. Sensing is based on the principle of affinity viscometry with a sensoric assay, which is separated by a semipermeable membrane from the tissue. Outer dimensions of the otherwise hermetically sealed biosensor system were 39 × 49 × 16 mm. The test system was implanted into cattle in a subcutaneous position without running it. After 17 months, the device was explanted and analyzed by comparing it with unexposed chips and systems. Investigations focused on the MEMS chip using SEM, TEM, and elemental analysis by EDX mapping. The sensor chip turned out to be uncorroded and no diminishing of the topmost passivation layer could be determined, which contrasts remarkably with previous results on CMOS biosensors. The negligible corrosive attack is understood to be a side effect of the semipermeable membrane separating the assay from the tissue. It is concluded that the separation has enabled a prolonged biostability of the chip, which will be of relevance for biosensor implants in general.

## 1. Introduction

Microelectronic chips as prepared by semiconductor technology are increasingly used in sensor systems for the detection of biomolecules [1,2,3,4]. Recently introduced microchips do not only expose simple metal electrodes, such as in cardiac pacemakers or neuro stimulators, but more complex semiconductor surfaces come into contact with body tissue or stroma. Technological surfaces are then subjected to the complex of foreign body reactions including macrophages, protein adsorption, innate immune responses, and fibrous encapsulation [5,6], which may disable functionality. In particular, in bioelectronics, i.e., the use of microelectronics in life sciences, the question of material and functional stability of the interface between semiconductors and the biological microenvironment arises [7,8,9]. Various investigations have dealt with the interaction of biological sources with surfaces of microelectronic chips, e.g., Ref. [10,11,12,13,14,15], but only a limited number were performed under *in vivo* conditions [16,17,18]. CMOS technology (complementary metal–oxide–semiconductor) is the dominating chip architecture, and whenever the *in vivo* corrosion of CMOS layers was investigated, severe loss rates on the order of 50–100 nm per month or more were determined [16,18].

Different transducing principles are being tested for continuous glucose monitoring in blood or stroma for *in vivo* medical diagnosis. Next to optical sensor systems [19,20,21,22,23], microelectromechanical systems (MEMS) were also developed to derive glucose variations from the viscosity changes of an affinity assay [24]. The biochemical assay consists of a solution of the glucose polymer dextran and the receptor molecule concanavalin A (ConA) [25,26]. The assay is separated from the stroma by a semipermeable membrane, through which glucose may pass and interact with the macromolecule network of ConA and dextran molecules. Measuring accuracies in the 1% range were demonstrated for glucose concentrations *c_g_* in laboratory studies using this sensor principle [27].

In the present work, the biostability of the CMOS/BiCMOS (bipolar CMOS) sensor chip was investigated. An encapsulation of the chip into a silicone housing has been developed previously [28]. The implantable system encompasses all functional components, but during the test the device stayed in a passive idle mode and was not operated as glucose monitor. The aim of this study was to test the biostability of the materials exposed, in particular, of the MEMS chip and its surface passivation, which both were in contact with the surrounding stroma. The restriction to a materials science study was performed in order to thoroughly understand one possible failure mechanism before bringing the sensor to a full operational *in vivo* test. Investigations were performed by electron microscopic and analytical techniques to identify possible differences between the implanted chip and an unstressed reference chip. The examination indicates that the semipermeable membrane, which primarily restrains the assay to the sensor cavity, also has a protective effect by withholding corrosive agents from the stroma.

## 2. Materials and Methods

**Figure 1:**
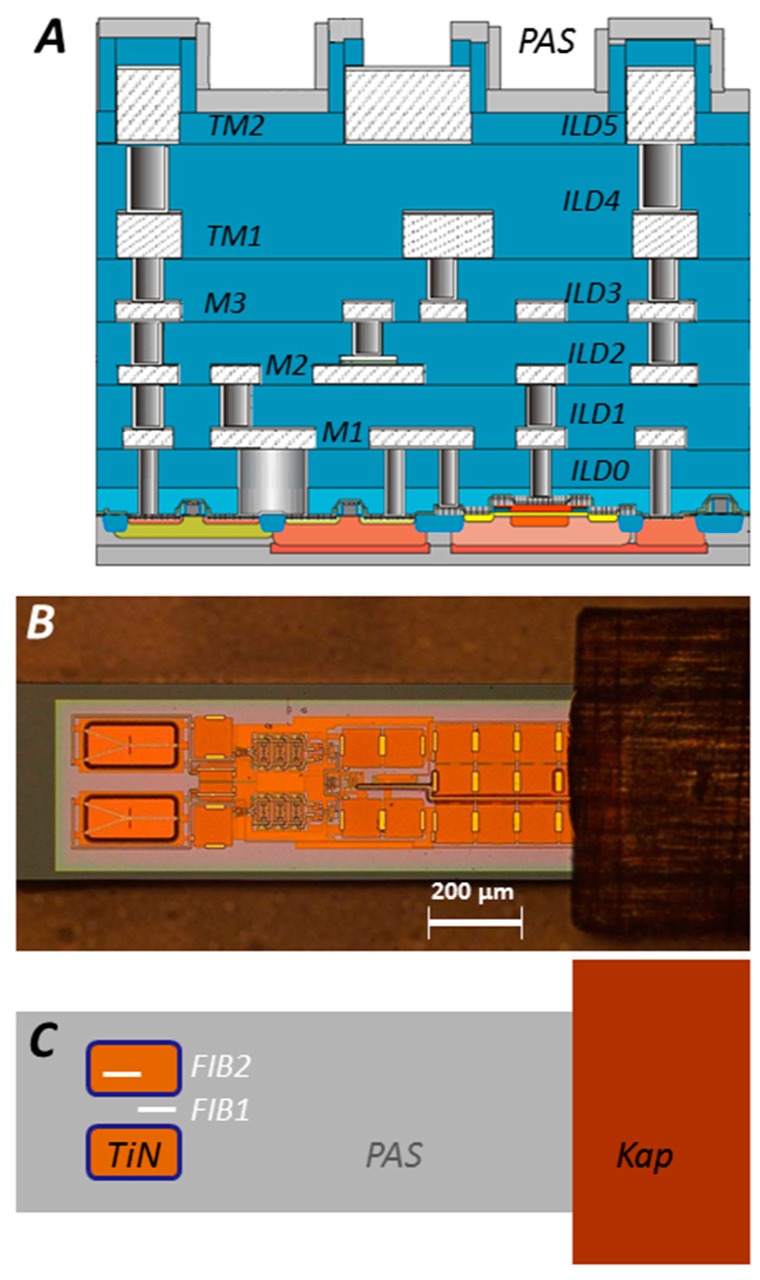
(**A**) Chip architecture of IHP 0.25 µm SGB25V technology as prepared on 200 mm CZ–Si wafers; (**B**) Optical microscope picture of fully processed glucose sensor chip of outer dimensions 1300 × 360 × 150 µm. The chip combines the measuring area with a TiN ground plate, frequency dividers, a phase frequency detector, temperature sensor, and bond pads for connecting to a flexible connection cable; (**C**) Schematic overview of chip material surfaces. Grey represents the SiON passivation layer PAS, blue flanks are SiO_2_ of the microelectromechanical system (MEMS) cavity, brown symbolizes the TiN MEMS ground plate and dark brown stands for the Kapton connection cable. FIB1 and FIB2 positions are indicated as white bars, from which lamella for transmission electron microscopy (TEM) were taken.

Sensor chips were prepared using IHP’s proprietary 0.25 µm SGB25V technology [4,29], the architecture of which is schematically given in Figure 1A. Its back end of line (BEoL) stack encompasses three metal layers M1–M3 and two top-metal layers TM1 & TM2 that are vertically connected by W plugs and which are all embedded in isolating SiO_2_ layers forming the interlayer dielectrics ILD0–5. Planar metal layers are essentially built up from Al:3%Cu with its bottom and top formed by antidiffusion Ti/TiN films of some 10 nm thickness. The full BEoL stack exhibits a thickness of about 15 µm and its topmost passivation layer PAS is formed from 400 nm silicon oxynitride SiON [27]. MEMS cantilever and ground plate were prepared from the bottom TiN of M3 and top TiN of M1, respectively. The passivation and MEMS TiN layers had the main contact with the biological microenvironment, and it was the TiN and PAS layer stability which had to be critically investigated in this study.

Sensor chips of an initial 750 µm thickness were prepared on 200 mm CZ–Si wafers that were thinned down to 150 µm after processing and chemically etched in order to release the TiN-made MEMS beams from the surrounding isolating dielectric. The last cleaning solution has to be dispelled by liquid CO_2_ in a critical point drying (CPD) step to avoid static friction (stiction) of the beam to the ground plate. In addition, sensor dies have to be separated from the wafer by a laser-assisted cutting process [30] and gold stud bumps have to be placed upon the three bond pads for *V_dd_*, *V_ctrl_*, and *GND* to enable the electrical connection with a flexible connection cable made from Kapton via a flip-chip bonding process (Figure 1B). The schematics in Figure 1C show the different materials exposed to the biological microenvironment.

The obtained chip-with-flex modules were glued into a cooling body, also fabricated from CZ–Si, for dissipating the heat produced during one measurement cycle [27]. A measurement chamber with a free volume of about 1 µL was established by gluing the semipermeable membrane with a cut-off of 2.2 nm onto the cooling body [31]. The biochemical assay (precise composition see [27,32]) was enclosed within the measurement chamber in subsequent filling and sealing steps. 

The obtained sensor probe was connected to a printed circuit board (PCB) that also encompassed a microcontroller (TIMSP430) for measurement control, a radio module (ZL70321) for wireless data transmission in the approved 402–405 MHz MICS band (Medical Implant Communication Service), and an antenna. A battery developed for medical implants (Litronik LiS 3150M 1200 mAh) was stacked below the PCB. 

The sensor system was cleaned, dried, and pretreated with adhesion promoter before depositing about 10 µm of parylene C (out of the cyclophan dimer Di-Chloro-p-Xylene, Specialty Coating Systems) in a parylene coater Para Tech LabTop3000 (Paratech, Frankfort, IL, USA). The sensor probe and antenna were fixed with silicone glue (Nusil MED3-4013). The membrane was covered by a piece of Kapton tape to prevent silicone penetration into the sensor. The upper and lower surfaces of the PCB were subjected to oxygen plasma in order to improve the adhesion of PDMS (polydimethylsiloxane) to the surface. For the same reason, the surface was pretreated with silicone primer Nusil MED1-161, and implant components were positioned and aligned in a casting box. The latter was placed in a vacuum-tight chamber, casted with silicone MED-6015 (Nusil) using the pressure difference technique, removed from the vacuum, and cross-linked with PDMS at 50 °C in an oven. The Kapton tape was subsequently stripped off and the edges of the sensor silicon body were coated with silicone glue (Nusil MED-1000) to avoid sharp edges. Finally, the silicone glue was cross-linked again at 50 °C for several hours [28]. 

In total, six implants Imp1–Imp6 were processed in three generations with two samples for each and the degree of integration constantly increasing. For third-generation systems Imp5 and Imp6, the battery was also electrically connected to the PCB. Corrosion stability tests were carried out with Imp3 and Imp6 in vitro and *in vivo*, respectively; see Table 1 for an overview.

For implantation, the left side of the cattles’ neck was chosen directly above the *Pars cervicalis mi. trapezii*. An area of 10 × 10 cm was cleaned and prepared for the operation. For sedation of the animal (Holstein Friesian), 1.5 mL Xylariem 20 mg (20 mg/mL Xylazine, Ecuphar GmbH, Greifswald, Germany) was injected intramuscularly. Two perpendicular lines were marked on the skin to presage a right triangle along the incision line. Beforehand, local anesthesia was administered by using 10 mL Isocain (20 mg/mL Procain-hydrochloride and 0.025 mg/mL Epinephrine, Selectavet Dr. Otto Fischer GmbH, Weyarn-Holzolling, Germany). The skin was cut along the marked lines and mobilized to place the implant into the created pouch. 

**Figure 2:**
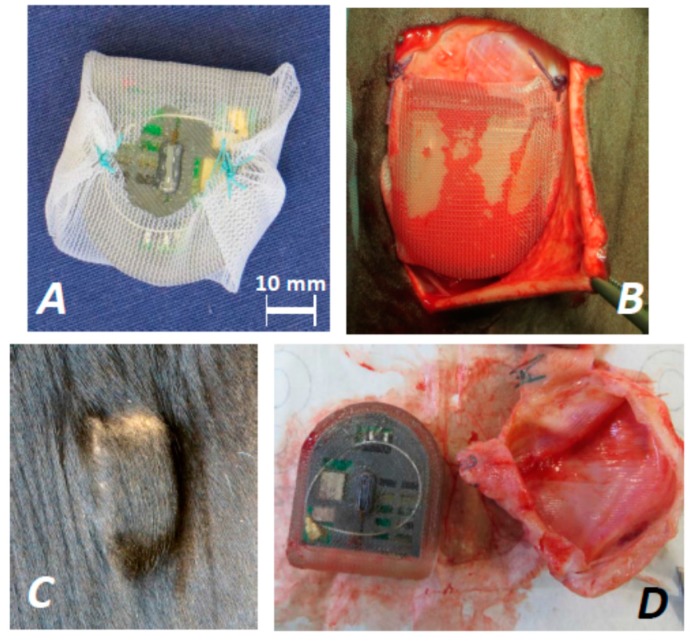
(**A**) The silicone-encapsulated implant Imp6 was wrapped in a surgical mesh in order to avoid dislocation during the course of experiment; (**B**) The implant was placed under the skin of cattle and fixed with suture material; (**C**) Implantation site after 3 months, showing no signs of inflammation; (**D**) Explanted sample Imp6 after 17 months of *in vivo* exposure to cattle stroma. The implant was completely overgrown by foreign body granuloma.

The implant had to be prepared by disinfecting it in isopropanol, dipping it in sterilized isotone saline, and wrapping it with surgical mesh to avoid its dislocation (Figure 2A). Subsequently, the sensor implant Imp6 was placed under the skin and fixed with suture material (Marlin EP6, Catgut GmbH, Markneukirchen, Germany) at the corners (Figure 2B). The skin was adapted and the wound was closed by using Polyester (EP6). No additional medical treatment was necessary. After seven days, the stitches were removed and the healing process was controlled regularly. (The procedures for implantation were carried out in accordance with the German Animal Protection regulations and were approved by the relevant authorities of the State Mecklenburg-Vorpommern, Germany (Landesamt fuer Landwirtschaft, Lebensmittelsicherheit und Fischereiwesen Mecklenburg-Vorpommern, Germany; permission no. 7221.3-1-049/14.) Three months later, the implantation site appeared normal and well cured, showing no indication of inflammation; see Figure 2C. After 17 months in cattle, the biosensor system was explanted (Figure 2D). The implant was autoclaved (MELAG Type 17) for 50 min at 1 bar and 120 °C, which was performed to avoid any contamination of the analytical equipment; afterwards, it showed essentially the same appearance as before implantation. 

The disassembling of samples Imp3, Imp5, and Imp6 for microscopic inspection was started by cutting the sensor probe from the top of the encapsulated device and cleaning them with a razor blade. The sensor probe was opened by uninstalling the window plate, including the semipermeable membrane, from the cooling body in order to allow the inspection of the sensor chip by optical and electron microscopy. After taking off the sensor probe, the system PCB was uncovered by removing all silicone mechanically with a razor blade, tweezers, and wooden toothpicks, which was carried out very carefully to avoid mechanical scraping of the PCB. 

Focused Ion Beam (FIB) with a Ga beam was used to prepare lamellae of 100–200 nm thickness for investigating the microstructure of sensor chips by transmission electron microscopy (TEM). Lamellae were also prepared from a sensor chip that has only been integrated into sensor probe GS21, which was filled with the biochemical assay, but which was not introduced into an implantable device, see Table 1. Investigations of the lamellae aimed at elucidating the layer architecture and allow for a comparison between exposed and unexposed sensor chips. 

Scanning electron micrographs (SEM) were taken with a ZEISS Merlin. For energy-dispersive X-ray spectroscopy (EDX) a ZEISS EVO25 was used, which was equipped with an EDX Genesis System and, in most cases, operated at an accelerating voltage of 10 keV. EDX maps of selected elements like Si, O, N, Cl, Ca, Na, Ti, etc., in SEM configuration were recorded after identifying energy regions of interest in X-ray emission spectra—for instance, 4.4 < *E* < 4.7 keV for the Ti*K* line, etc.—and counting area-resolved scattering events in these energy windows only. Some of the maps were sampled by 50 or more runs due to small concentrations of elements in certain areas. Integration times amounted to 1 h or more, causing small lateral drifts in some maps because of the charging effects of nonconducting materials. (Scanning) Transmission electron microscopy ((S)TEM) and EDX investigations of FIB lamellae were performed using a FEI Tecnai Osiris operated at 200 kV. The full spectral information was recorded in the case of EDX map measurements in STEM mode. 

## 3. Results

First, the results of implant Imp3 will be presented, which was simply submerged in isotonic saline for 24 months. Remarkably, no corrosion effects were visible through the transparent silicone encapsulation before the device was disassembled. For a detailed investigation the whole system had to be disjoined, for the purpose of which all polymer components were detached.

**Figure 3:**
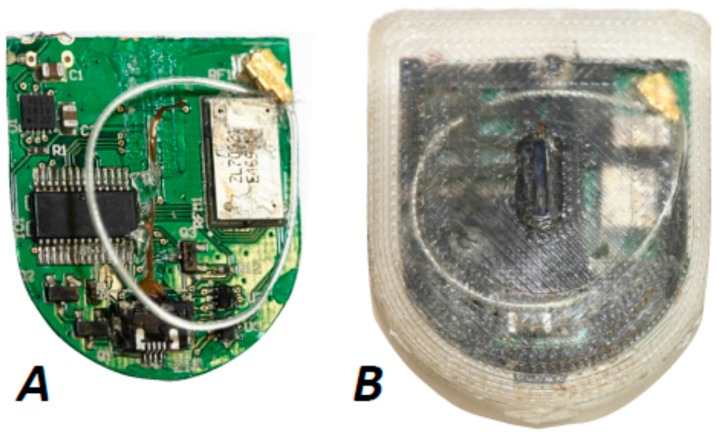
(**A**) After 24 months in isotonic saline the implant Imp3 was disassembled and inspected by optical microscopy; (**B**) The cleaned and sterilized Imp6 appeared fully intact after explantation when judged from its outer appearance.

Even after uncovering, no corrosion of the PCB could be detected with an optical microscope; see Figure 3A. Further investigations of the PCB by EDX mapping was expected to reveal the presence of relevant elements like Na and Cl, which are the most abundant ions in mammalian extracellular fluid with concentrations above 100 mM [33]. Both elements were indeed detected covering the PCB in small densities (maps not shown here), albeit that their quantification was difficult. The supplier of the silicone (Nusil MED-6015) used for the housing only indicates a transmission rate for water vapor of 62.2 g m^−2^ day^−1^, but specifies no diffusion coefficients for ions from electrolytes [34]. However, Na and Cl ions can be expected to have diffused from the surrounding saline onto the PCB. It could be concluded that no corrosion of the PCB or outer components of the electronic system of Imp3 could be detected by optical microscopy or EDX mapping.

The fully implanted system Imp6 was disassembled in the same way as Imp3 after explantation and sterilization (Figure 3B). However, whereas the battery of Imp3 had a residual voltage of 3.00 V, the one of Imp6 amounted to 2 mV only. This is understood from the fact that the battery of Imp6 was electrically connected to the PCB and that the Imp3 battery remained unplugged. Obviously, small leakage currents to other system components discharged the battery during the 17 month experiment, although the sensor system was not operated.

**Figure 4:**
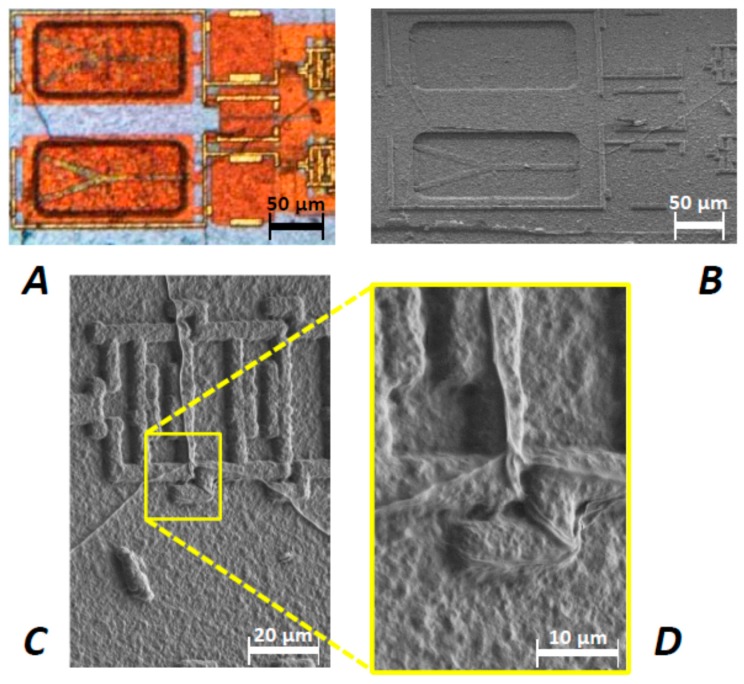
(**A**) Optical micrograph of Imp6 chip after explantation with dried-up biomolecular layer covering it. Essentially, the latter appears transparent, albeit that some granular inclusions restrict the optical resolution of MEMS details like the TiN beam etc.; (**B**) In SEM, the layer appears like a granular layer deposited on the chip; (**C**,**D**) Detailed views of micro fibrils on the chip surface.

Opening the sensor probe of the implanted system Imp6 revealed an interesting view on the MEMS chip. Figure 4A–D display the micrographs obtained by optical and scanning electron microscopy. The sensor chip is seen to be covered by a thin biomolecular layer that appears like a dried-up suspension of organic components (Figure 4A,B). The film probably consists of components of the sensoric liquid ConA and Dextran. It is also possible that some small-sized components of the cow stroma passed through the semipermeable membrane.

An interesting phenomenon relates to the appearance of fibril structures across the biomolecular layer with a diameter of about 250–400 nm (Figure 4C,D). It could be possible that the structures floated above the MEMS ground plate in the measuring cavity during the trial. These biomolecular deposits may be formed from lipids like cholesterol or proteins like albumin. The complex 3D structures might have formed inside the cavity from structural protein monomers, which passed the semipermeable membrane individually. This would, however, pose the constraint to the monomers that their extension must not exceed the cut-off diameter of the membrane, which amounted to 2.2 nm.

**Figure 5:**
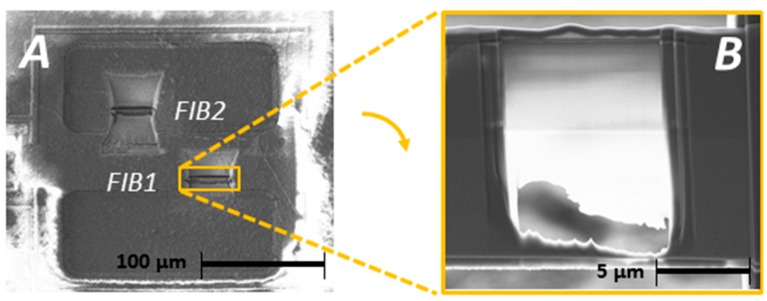
(**A**) Positions FIB1 and FIB2 for the extraction of lamellae from the MEMS area of exposed and unexposed sensor chips; (**B**) SEM micrograph of an extracted chip lamella that was selected for EDX mapping.

We will now discuss the architecture or layer structure of exposed sensor chips as inspected by TEM. About 150 nm thin FIB lamellae were extracted from the bottom of the MEMS cavities and passivated areas (Figure 1A and Figure 5A). Before dicing the lamellae from the chip, a ca. 600 nm thin carbon layer was deposited by ion-induced deposition. This C layer had to protect the chip surface in order to avoid damaging near-surface regions with the Ga ion beam.

**Figure 6:**
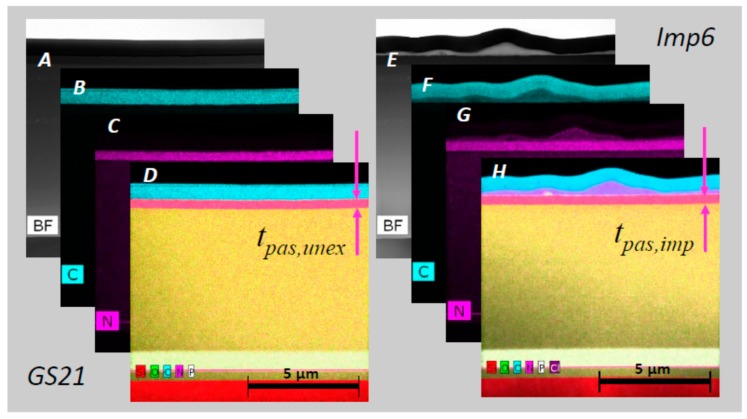
TEM micrographs and EDX maps of focused ion beam (FIB) lamellae from GS21 and Imp6 taken from the FIB1 position. (**A**) TEM bright field view of the unexposed GS21 reference chip; (**B**,**C**) EDX maps of main elements carbon and nitrogen; (**D**) Superposition of all elements mapped in the lamella; (**E**–**H**) Imp6 lamella exhibiting a biomolecular layer on top given in the same sequence as in (**A**–**D**).

Lamellae were taken from sensor chips integrated in the sensor probe GS21 as well as from full biosensor implants Imp3 and Imp6. Figure 5B shows a SEM micrograph of a FIB lamella as extracted from the PAS layer next to the sensor cavity of GS21. The organic composition of the top layer was verified by EDX mapping the elements C, N, Si, O, Ti, and P. Figure 6A–D combine the TEM bright field view with the main elements carbon and nitrogen and other elements occurring in the lamella. A thorough inspection reveals that the stack had met the specification of IHP SGB25V technology; in particular, the topmost SiON passivation shows a thickness of 375 ± 20 nm, which is thus sufficiently close to the intended value of 400 nm.

**Figure 7:**
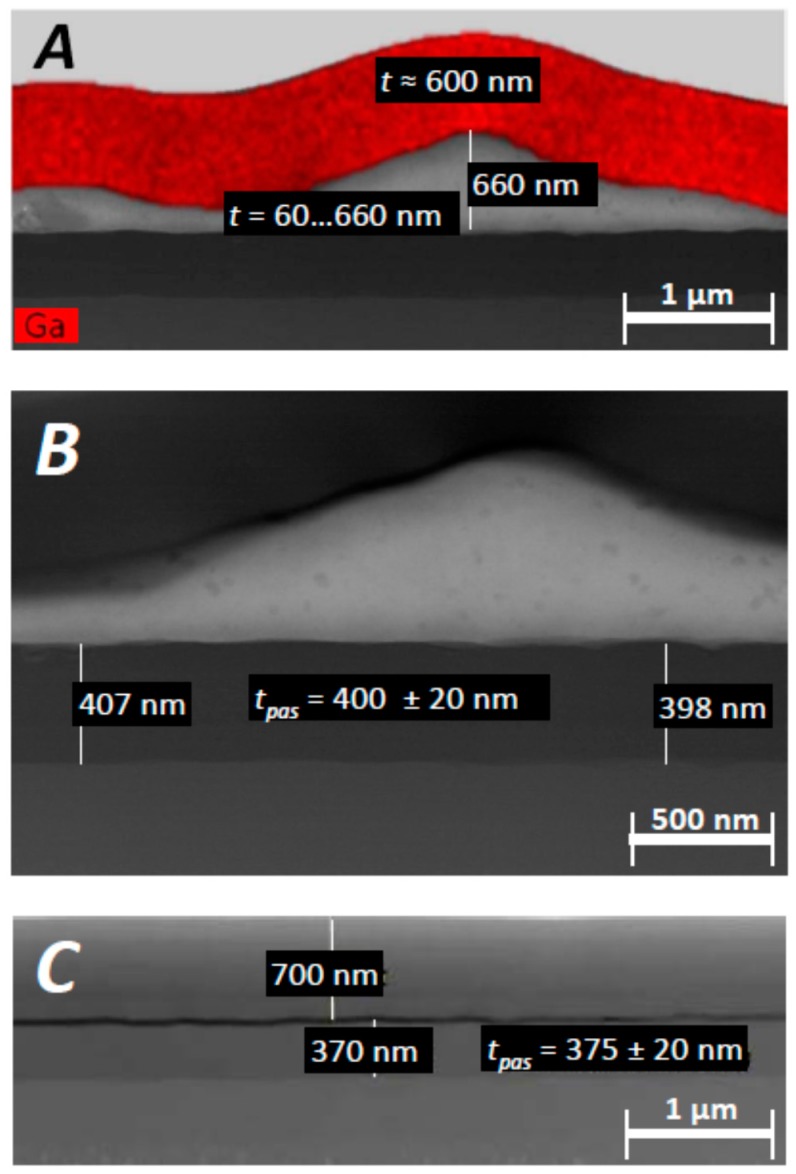
Thickness measurement of the topmost layers from TEM micrographs of Imp6 and the GS21 reference chip. (**A**) Measurement of the carbon protective layer (with highlighted Ga atoms) and the biomolecular layer lying underneath; (**B**) Dimensions of the SiON passivation layer under the biolayer; (**C**) Reference measurement of the protective layer and the SiON layer of unexposed MEMS chip from sensor probe GS21.

Another lamella that was extracted from the FIB1 position of the Imp6 sensor chip yielded similar results. A TEM image in bright field mode gives an overview of the obtained layer stack, shown in Figure 6E, and EDX maps are shown in Figure 6F–H. The biomolecular layer mentioned above is realized on top of the Imp6 lamella underneath the protective carbon layer. The latter is interspersed by Ga atoms, from an ion beam with which the FIB lamellae were prepared. The average thickness of that layer was on the order of 600 nm. Like the protective C layer, the layer thickness was derived from TEM micrographs and was found to lie in the 60–600 nm range; see Figure 7A.

The rest of the Imp6 layer stacking matched the layer architecture of the reference GS21 chip. No reduction of the top SiON layer is visible for Imp6 in comparison to GS21. Therefore, it can be concluded that the exposed Imp6 chip shows no layer reduction; rather, the thickness of the SiON layer perfectly met the technologically intended value of 400 ± 20 nm as shown in Figure 7B. This is a remarkable and unexpected result, because previous *in vivo* investigations of stroma-exposed sensor chips revealed diminishing rates on the order of 50 nm per month in human tissue [18]. If the same mechanisms would have affected the implant sensor chip, the PAS layer with a thickness of 400 nm would have been fully delaminated, which would have paved the way for corrosive attacking of the underlying SiO_2_ layers. Interlayer dielectrics (ILD) from SiO_2_ were shown in previous investigations to suffer much more strongly from biocorrosion than were the SiON passivation layers [16]. Leaving the passivation layer intact can thus be considered a key enabling factor for establishing a sufficiently long operation time of microelectronic chips in body implants.

The fact that the previously determined corrosion velocity of the passivation layer was not observed in the implanted CMOS/BiCMOS chip might be explained by the effect of the semipermeable membrane. With a cut-off diameter of 2.2 nm, the membrane separates not only the biochemical assay, but also the sensor chip from the stroma. It can be concluded that the main substances of cattle stroma, which would cause chip corrosion, are larger molecules or macrophages with diffusion diameters in excess of the membrane cut-off. The membrane thus effectively protects the chip by blocking these corrosive components.

## 4. Conclusions

To summarize, a fully integrated, but unoperated biosensor system was implanted in cattle for about 1.5 years in order to study the biostability of system components. Silicone was applied to house the sensor system, which interacted with the tissue via a semipermeable membrane allowing the passage of low-molecular-weight metabolites. Corrosive defects were only observed at the PCB soldering joints to the battery that were probably caused by diffused water at the silicone–PCB interface. Microfibril-like structures were detected by SEM that were formed during the implantation time in the sensor cavity behind the semipermeable membrane. Most remarkably, etch rates of the exposed sensor chip were so small that no reliable value, significantly different from zero, could be derived for PAS or ILD layers. The effect contrasts with previous investigations, where unprotected microchips were exposed to stroma and etching rates on the order of 50 nm per month were observed. The effect is understood to be due to the protective function of the semipermeable membrane dispelling corrosive tissue components out of the sensor cavity. This result is of general relevance for the use of CMOS/BiCMOS chips in implants and other biomedical devices. It suggests that the biostability of microelectronic chips can be significantly increased by using semipermeable membranes with an exclusion (cut-off) diameter in the nm range, protecting the chip from corrosive tissue components.

## Figures and Tables

**Table 1:** Samples investigated and their exposition to corrosive environments (PCB stands for printed circuit board).

Device	Degree of Integration	Exposure
GS21	sensor–probe integrated MEMS chip	None
Imp3	battery unplugged	>2 years in isotone saline
Imp6	battery connected to PCB	17 months *in vivo* and hv sterilization
